# Trends in Mortality From Novel Psychoactive Substances as “Legal Highs”: Gender Differences in Manner of Death and Implications for Risk Differences for Women

**DOI:** 10.3389/fpsyt.2022.890840

**Published:** 2022-04-21

**Authors:** Lucy Webb, Xin Shi, Christine Goodair, Survjit Cheeta

**Affiliations:** ^1^Department of Nursing, Manchester Metropolitan University, Manchester, United Kingdom; ^2^School of Maths and Information Science, Shangdong Technology and Business University, Yantai, China; ^3^Business School, All Saints Campus, Manchester Metropolitan University, Manchester, United Kingdom; ^4^St George's Hospital Medical School, University of London, London, United Kingdom; ^5^Department of Life Sciences, Brunel University, London, United Kingdom

**Keywords:** novel psychiatric substances, women, drug mortality, drug-related death, female mortality

## Abstract

**Introduction:**

This study aimed to examine drug-related deaths in the UK in which novel psychoactive substances (NPS) are an implicated substance, and to focus on female deaths in comparison with male deaths. While male overdoses dominate epidemiological statistics, there is an increase in female drug-related deaths and a narrowing of the gap between gender mortality rates which is to date unexplained.

**Method:**

This study analyzed data from the National Programme for Substance Abuse Deaths (NPSAD) database that records drug-related deaths in the UK from coronial records. A dataset was constructed using parameters to capture all drug-related cases during the period 2007–2017 when NPS were legal and highly available in the UK, in order to capture deaths recorded among both regular and occasional drug users, and to include all cases recorded during that period regardless of NPS status in order to make comparisons. The final dataset comprised 10,159 cases, with 456 NPS-related deaths. Data for NPS and non-NPS were compared, and comparisons were made between cohorts by gender. The dataset also includes coronial narrative notes which allowed a qualitative analysis of NPS female deaths to add contextual explanation.

**Results:**

The proportion of male NPS deaths is significantly higher than that for female NPS deaths but does not reflect the generalized difference between male and female drug-related mortality of this period studied. Demographic and outcome data by gender difference were significant for all drug-related deaths, but not for NPS-only deaths, indicating a greater homogeneity among NPS deaths by gender. Older women using NPS were more likely to have methadone or diazepam as another drug implicated and have established histories of drug misuse.

**Conclusion:**

Where NPS have been used, differences in drug death profiles are less likely to be accounted for by gender than other demographic or behavioral differences more typically found in opiate deaths. The social and health problems of older women may be key characteristics that differentiate female deaths from male deaths. These findings also support evidence of increasing uptake of NPS among older established drug users that adds further risk to polydrug use.

## Introduction

Most acute drug-related deaths globally are among males more than females (1). However, in the United Kingdom (UK) and elsewhere the gap between male and female rates of acute drug-related deaths is closing. Acute drug-related deaths for the purposes of this study are those associated with overdose and acute intoxication rather than from secondary related diseases such as hepatitis, cardiac conditions and obstructive pulmonary disease.

Evidence for drug-related mortality causes and circumstances often overshadows female mortality due to the disproportionate ratio between genders, and the not so uncommon practice of “controlling for” gender, i.e., excluding female deaths or not examining gender differences in drug mortality (2). Opiates in overdose also dominate as the main cause of death in population statistics, again overshadowing circumstantial and secondary causes. Indeed, controlling for gender itself suggests a different profile of female drug-related death, as might controlling for, or at least exploring in more detail, overdose deaths where opiates are implicated, which could reveal commonly overshadowed factors associated with drug deaths.

While male overdoses dominate epidemiological statistics, there is an increase in female drug-related deaths and a narrowing of the gap between gender mortality rates (1), and this is to date unexplained. In Scotland, which currently records the highest rate of drug-related deaths (DRDs) per capita in Europe (3), the gap forDRDs between genders has been narrowing since 2016/8, with a reported 212% increase in female DRDs against a 75% increase in male DRDs (4). The European Monitoring Centre for Drugs and Drug Addiction (EMCDDA) indicates that the ratio difference between girls and boys aged 15–16 has been narrowing in Europe (5), and drug deaths among women between 30 and 64 has increased by 260% in the United States (6).

Factors implicated in the rise are suggested to be greater accessibility of opiates, polydrug misuse and polypharmacy (prescribed medications) (7), and impacts from psychosocial and socio-economic pressures that disproportionately affect women (8). The increase in fentanyl prescribing in the United States is likely to be skewing the global rates of opiate use and mortality among women (9), but other national data indicate this may be symptomatic of other female-specific trends, and that opiate prescription availability is merely highlighting this issue. Tweed et al. (4) suggest that the aging cohort of drug users, accompanied by increasing physical and mental health problems, may affect women's vulnerability to DRD more than men, as too, they suggest, might changing patterns of drug use, with deaths with methadone implicated being slightly higher in female DRD than male DRD in Scotland. The NPSAD surveillance programme has recorded gender differences in types of drug implicated in deaths across England and Northern Ireland, showing male deaths to be most associated with heroin, and females deaths most associated with other opiates and opioid analgesics (10). In comparison with male DRDs, female DRDs proportionately involved more antidepressants, hypnotics and sedatives, methadone and antiepileptics (i.e., gabapentin), and more likely judged to be “suicide” or “undetermined” than male deaths (10).

The rise in female substance use and mortality has coincided with the rise of novel psychoactive substances (NPS) and increased availability of these substances across Europe and, increasingly, globally. These substances, often known as designer drugs or sometimes legal highs, may mimic the effects of illegal substances while evading national drug laws. Wastewater analysis indicates that NPS use has grown in Europe and increasingly around the world (11). Globally, nation states have changed legal systems to take account of the manufacturers' ability to tweak synthetic compounds, however, the illegality of the substances is proving to be little deterrent to use. Availability has switched from open retailing of legal highs to darknet ordering (12) with synthetic cannabinoids proving to be the most popular NPS, predicted to take over international drugs markets (13, 14), making them one of the most easily accessible psychoactive substances in the illicit drugs market.

This study aimed to examine drug-related deaths in the UK in which NPS is an implicated substance, and to focus on female deaths in comparison with male deaths. We are particularly interested in not only examining variables related to cause of death and substances implicated but to examine the circumstances of death, using the narrative evidence from coroners' reports. In this way, we aim to interrogate qualitatively the differences between male and female drug-related deaths and so explore explanations for differences in behavior and cause and manner of death.

## Methods

### Data Source

The National Programme on Substance Abuse Deaths (NPSAD) collates coronial, Procurator Fiscal and Scottish Crime and Drug Enforcement Agency reports on deaths in the UK where a psychoactive substance is implicated or a controlled drug is present at post mortem. The NPSAD programme was established to provide surveillance data to inform policymakers, commissioners and service providers to emerging drug use patterns and to support prevention initiatives in tackling drug-related deaths in the UK. Established in 1997, the NPSAD database has maintained this record with coverage across England, Wales, Northern Ireland, Scotland (until 2011) and the Crown dependencies of Guernsey, Jersey, and the Isle of Man. Data recorded for each case includes demographics, jurisdiction, specific location of death, drugs present post mortem (and concentrations), drugs implicated (i.e., found at the scene and drugs at post mortem considered to be implicated in the death), medically determined cause of death (ICD10 codes), coroner's verdict, prescribed psychoactive medications and mental health status, as determined by additional information from the reporting authority, prescribed medication and textual analysis of narrative reporting. The database also contains notes from coroners' narrative reports giving details of the circumstances of the death as obtained from police and witness reports.

### The Dataset

The dataset was extracted from the NPSAD database. We were particularly interested in constructing parameters to capture all drug-related cases during the period when NPS were legal and therefore highly available in the UK to both regular and “recreational” or occasional drug users. Date parameters were therefore set to include all NPS deaths over a 10-year period up to the implementation of the Psychoactive Substances Act (2016) (2007–2016) and to include all cases recorded during that period regardless of NPS status. The dataset includes drug deaths coverage of approximately 75% of UK regions, reporting inquest cases where one or more psychoactive substances is directly implicated in the death (found at post mortem at significant levels), or where a drug listed in the Misuse of Drugs Act (1971) is found to have been used at the scene of death.

Cases were excluded where no drug was implicated or present or where only alcohol was implicated or present. This excluded deaths that were clearly not associated with the intake of a psychoactive substance, such as death occurring during drug dealing activity (i.e., inter-gang shooting) or lawful killing, but included trauma associated with drug-impaired judgement such as road traffic accidents, drowning or suicide. Cases were retained if most of the relevant data were available, with missing values for a small proportion of cases for some demographic variables, living arrangements, and if known to be a drug user. The final dataset comprised 10,159 cases, with 456 NPS-related deaths.

### Methodology and Analysis

Categorical demographic variables were collapsed into bivariates, including gender. The ethnicity variable contained a large proportion of missing values or unreliable classification and was therefore excluded from the analysis.

Cases of NPS deaths were compared to cases where no NPS substances were implicated or found at post mortem. This was an observational study with analysis based on proportions and ratios to give point estimates. Measures of association between variables for non-parametric distributed data were applied using Kendall's Tau-b. Regression analysis was applied to confirm levels of association. Gender comparisons for age and mental health history were conducted using one-way ANOVA. Analysis was performed using SPSS 27.

## Results

### Profile of Cases

Table 1 shows the profile of cases for NPS and non-NPS deaths by relevant demographic variables. There are significant differences across democratic variables and outcomes except where a person is or is not known to be a drug user.

**Table 1:** Profile of cases by NPS death status^*†*^.

**Category**	**Bi-variate**	**NPS implicated or at PM *N* (%)**	**No NPS implicated or at PM *N* (%)**	**Kendall tau-b** **(OR, CI 95%)**
Gender	Male	389 (85.3)	7,313 (75.4)	5.62^**^ (1.90, 1.5–2.5)
	Female	67 (14.7)	2,390 (24.6)	
Age	≤38	315 (69.1)	4,495 (46.4)	9.34^***^ (2.59, 2.11–3.16)
	>38	141 (30.9)	5,201 (53.6)	
Living arrangements	Stable	393 (95.6)	7,959 (91.0)	4.32^**^ (2.17, 1.34–3.50)
	Unstable	18 (4.4)	790 (9.0)	
Employment	Employed	198 (48.9)	2,805 (31.9)	−6.40^**^ (0.49, 0.41–0.60)
	Unemployed (inc. retired, student, other)	207 (51.1)	5,985 (68.1)	
Place of death (public place vs. home)	Public (including hospital)	52 (14.9)	750 (9.7)	−2.66^*^ (0.61, 0.45–0.83)
	At home (inc. all domestic settings)	298 (85.1)	6,998 (90.3)	
Known drug user (excludes “not known”)	Yes	233 (74.7)	5,554 (74.2)	0.19 (1.03, 0.79–1.33)
	No	79 (25.3)	1,931 (25.8)	

† *Excludes missing values*.

* *p < 0.05*,

** *p < 0.01*,

*** *p < 0.001*.

The proportion of male NPS deaths is significantly higher than that for female NPS deaths (5.1–2.7%). The association between NPS and gender is statistically significant (Kendall tau-b *p* < 0.001). Odds ratios indicate that NPS death risk for males is 1.897 times more than that for females (OR = 1.897, 95%CI = 1.46–2.47). This gap does not reflect the generalized difference between male and female drug-related mortality during this period which would more typically be around 4:1 up to 2016 (15), and represents a narrowing of rates by gender where NPS are implicated.

Comparison between NPS and non-NPS variables indicates that NPS cases are significantly more likely to be employed (or be a student/pupil) than non-NPS cases (*p* < 0.01) and have stable living arrangements (*p* < 0.01). Odds ratios indicate that, while most drug-related deaths occur in a home environment, NPS deaths are less likely to occur in a home environment in comparison with non-NPS deaths (OR = 0.61, 95% CI = 0.45–0.83). The association between NPS and place of death is statistically significant (Kendall tau-b, −2.66, *p* < 0.05), but the relationship is weak.

### Gender

Gender comparisons for the whole cohort show significant differences across demographics and outcomes (Table 2), but no significant differences were found when analysis was restricted to cases in which an NPS is implicated in the death. Among NPS cases, 68.7% females were 38 years or under, in comparison with 69.2% males; 96% of females had stable living arrangements in comparison with 95% of males; known drug user recorded as 72.5% female and 75.0% male. Only unemployment (female = 60.7%; male = 49.6%) and death in a public place (female = 8.7%; male = 15.8%) indicated any notable gender difference without significance. This finding from comparison between whole cohort and NPS-only cases suggests a different profile of drug-related deaths and a homogenisation of drug-taking behavior between genders when NPS are involved. This narrowing is highlighted by restricting analysis by drug type; when the majority of opiate-related deaths are reduced.

**Table 2:** Whole cohort demographic and outcomes by gender^*†*^.

**Category**	**Bi-variate**	**Females *N* (%)**	**Males *N* (%)**	**Kendall tau-b (OR, CI 95%)**
Age	≤38	995 (40.5)	3,815 (49.6)	7.85^**^ (1.4, 1.3–1.6)
	>38	1,459 (59.5)	3,885 (50.4)	
Living arrangements	Stable	2,110 (95.0)	6,242 (90.0)	−8.4^**^ (4.8, 0.39–0.59)
	Unstable	112 (5.0)	696 (10.0)	
Employment	Employed	506 (23.0)	2,497 (35.7)	−11.7^**^ (0.54, 0.48–0.60)
	Unemployed (inc. retired, student, other)	1,691 (77.0)	4,501 (64.3)	
Place of death (public place vs. home)	Public (including hospital)	78 (4.1)	724 (11.7)	−12.3^**^ (0.32, 0.23–0.41)
	At home (inc. all domestic settings)	1,834 (95.9)	5,462 (88.3)	
Known drug user (excludes “not known”)	Yes	1,147 (62.6)	4,640 (77.8)	12.0^***^ (2.1, 1.8–2.3)
	No	686 (37.4)	1,324 (22.2)	

† *Excludes missing values*.

** *p < 0.01*,

*** *p < 0.001*.

### Gender Differences by Substance

Analysis of gender differences for the whole cohort showed significant higher female proportions for anti-depressants implicated and prescribed, hypnotics/sedatives implicated and prescribed, methadone implicated and diazepam prescribed (See Table 3). This reflects contemporary and recent findings for substances associated with female DRDs. However, an exploration of NPS cases by gender showed no significant differences between gender proportions except for methadone implicated (females = 20.0%, males 9.0%, OR = 1.218, (95%CI = 1.02–1.45) (Kendall tau-b *p* < 0.025). There is also a small significant difference in implicated methadone ratio for females for the whole cohort (females = 24%, males = 22%) (Kendall tau-b *p* < 0.047), but this difference is more significant among NPS deaths (*p* < 0.025), showing that, where a NPS is used, methadone is more likely to also be used by females than males. Of female deaths with methadone implicated, nearly two thirds also had diazepam implicated, but only two NPS cases were prescribed diazepam, and only one case prescribed methadone. This also suggests that many women using NPS are more likely than men to also be sourcing illicit depressant/sedating medication.

**Table 3:** Whole cohort, substances implicated and prescribed by gender^*†*^.

**Substance implicated in death or prescribed**	**Females *N* (%)**	**Males *N* (%)**	**Kendall tau-b (OR, 95% CI)**
Antidepressants implicated	747 (30.4)	1,034 (13.4)	−16.54^**^ (0.36, 0.32–0.40)
Antidepressants prescribed	1,138 (46.3)	2,008 (26.1)	−17.61^**^ (0.41, 0.37–0.45)
Hypnotics/sedatives implicated	569 (23.2)	1,629 (21.2)	−2.07^*^ (0.89, 0.80–0.99)
Hypnotics/sedatives prescribed	781 (31.8)	1,561 (20.3)	−10.93^**^ (0.55, 0.49–0.60)
Methadone implicated	589 (24.0)	1,696 (22.0)	−1.99^*^ (0.90, 0.81–0.10)
Methadone prescribed	275 (11.2)	772 (10.0)	−1.62 (0.88, 0.76–1.02)
Diazepam implicated	379 (15.4)	1,234 (16.0)	0.71 (1.05, 0.92–1.19)
Diazepam prescribed	441 (17.9)	934 (12.1)	−6.76^**^ (0.63, 0.56–0.71)

† *Excludes missing values*.

* *p < 0.05*,

** *p < 0.01*.

Low numbers of NPS female cases reduces the reliability of analyses of several substances, including infrequently occurring substances. During this period of developing NPS use, up to their outlawing in the UK by 2016, designer benzodiazepines such as phenazepam, flubromazepam, diclazepam and etizolam were a growing phenomenon but low in rates of use in Europe (16). Our analysis shows very small numbers of cases where designer benzodiazepines were implicated in NPS deaths, and particularly few if any among female deaths.

### Age

Binomial age was based on the median age (38) for the whole cohort. For those 38 years old or under, the proportion of NPS deaths is more than twice than for those over 38 years. The association between NPS and age is statistically significant (Kendall tau-b = 9.34, *p* < 0.001). Odds ratios indicate that the NPS risk of death for those under 39 is 2.6 times more than that for non-NPS deaths (OR 2.59, 95% CI = 2.11–3.16). There was no significant difference in age at death by gender among NPS deaths (one-way ANOVA F=.152, *p* < 0.70) indicating that NPS use is more prevalent among younger people regardless of gender.

### Mental Health History

Known mental health history is included in the NPSAD database, derived from coronial reporting of additional information, prescribed medication or narrative analysis. These data are recorded in the database as separate variables for known history of anxiety, depression, paranoia, eating disorder, PTSD, bi-polar disorder, schizophrenia and psychosis. The strongest association with NPS deaths was psychosis (Kendall tau-b = −0.33, *p* < 0.02) with odds ratios indicating that risk of having a reported psychotic issue among NPS cases being 2 times more likely than for non-NPS cases (OR 0.43, 95% CI = 0.26–0.72). For the whole cohort, having a known mental health history was significantly more frequent among female deaths (26.1%) (16.3%) (Kendall tau-b = 0.11, *p* < 0.0001) but no significant gender difference among NPS-only cases (Kendall tau-b = −0.62, *p* < 0.14). The association between younger age and NPS may be one explanatory factor of lower mental health history incidence.

Mental illnesses were otherwise reported in similar proportions for NPS and non-NPS cases, with 4.6 and 3.1% of NPS and non-NPS cases with known anxiety, 10.3% and 11.1% of cases, respectively, with known histories of depression. There were 2.2 and 2.4% of NPS and non-NPS cases recorded as having schizophrenia and 0.9 and 1.0% of cases, respectively, with known histories of bi-polar disorder. Non-NPS cases recorded 0.2% (*n* = 18) with PTSD, with no cases among NPS cases. Low and absent numbers of psychosis, paranoia and eating disorder among female NPS deaths precluded gender comparisons.

## Narrative Data

The dataset provides coroners' verdicts and notes from coroners' narrative verdict which together give circumstantial detail on individual cases and allows a narrative analysis. This was conducted following the analysis of quantitative data providing an analysis focused on quantitative results to better explain these findings. This took the form of a thematic analysis of all female NPS cases that had narrative data (*n* = 55), using, firstly, a priori themes based on factors that gave circumstantial detail of the death, but otherwise open to emergent themes within this evidence. The variables included were age, cause of death and verdict, narrative reporting, implicated substances and prescribed medication.

Guided by the quantitative findings, we firstly examined cases of female NPS deaths where methadone and/or a benzodiazepine were implicated, particularly examining place and circumstances of death, coroner's verdict and indications of other health or social factors associated with the person and their death. Following this, contrasting cases were examined, that is, where methadone or benzodiazepines were not implicated in the death. This provided qualitative comparison to those female deaths that differed from male deaths.

### Female NPS Related Deaths Associated With Methadone and/or Benzodiazepines

Examination of the cases associated with methadone and/or diazepam demonstrate a difference in circumstances surrounding the death according to age. Women in their thirties and over tended to have associated physical and mental health histories and prescribed medications that were implicated in their deaths. These also often suggested a story of long-term drug use and social and health problems. For example, one woman aged 30 of no fixed abode died from a pulmonary embolism having taken heroin, ethylphenidate (piperidine) and diazepam. She had been in prison, had a history of chronic heroin use and was prescribed antidepressants, diazepam, and propranolol (beta-blockers). She had injected into her groin when found.

Another woman, aged 48, was found dead at a home run by a housing charity that aims to encourage homeless people into secure housing. The reports states “empty methadone bottles and a used needle were found at the scene”. The cause of death was recorded as natural causes due to chronic illness, cirrhosis of the liver and bronchitis. Toxicology reported a mixture of mephedrone with methadone and diazepam, neither of which were prescribed.

Among younger methadone or diazepam-related deaths, there were also some individuals with a history of problem drug use but also recreational use. A 28-year-old student with part time employment was reported to have been found at home; “dead on lounge floor by her estate agent” following “an evening with work colleagues on Saturday”. She had used alcohol, GBL, diazepam, oxazepam, temazepam and mephedrone. She was reported to have recently become depressed, drinking heavily and taking drugs. She was only prescribed antidepressants.

Another younger death example was a 24-year-old who lived with friends who was found dead from “accidental toxicity” having “been to a party during the night and taken drugs”. Drugs implicated in her death were 4-MEC (a cathinone), piperidine, cannabis, diazepam, mirtazapine and codeine. She was not prescribed any medication according to the report.

Clearly, polydrug use features in all these cases as such cases (with methadone and benzodiazepines) were selected for analysis, however the amount of drugs found implicated or at post mortem indicates, at best, recklessness among chronic drug users and naivety among those that could be described as “recreational” users. While there is a difference between implicated drugs (those causative of death) and those found at post mortem, the presence of multiple interactive and cumulative substances is commonly reported in polydrug deaths (17, 18). There are also no implied differences between these female deaths and male NPS-related deaths. However, our findings indicate a higher proportion of deaths among females where the drugs were prescribed. As the above example of the woman with no fixed abode and with a prison history, she had been prescribed diazepam and anti-depressants.

A 62-year-old woman who lived on her own and found dead at home had been prescribed olanzepine, duloxepine, diazepam and nitrazepam (all found present at post mortem), and had taken 4-MEC, mephedrone, methylone and ecstasy. She was known to use heroin, diagnosed with depression, ischaemic heart disease and had a stroke. She also had cognitive impairment, possibly due to her stroke. Her cause of death was recorded as cardiac atheroma, however, one may wonder what her state of mind had been on that evening of drug use.

For a 32-year-old, prescribed methadone was implicated in her death from hypoxia following polydrug use. The narrative notes stated:

“…before her death took a substantial quantity of drugs with her partner including heroin, cocaine and legal highs, and went to bed. Next day found gurgling and unrousable and taken to hospital. Died four days later showing no brain activity.”

This woman had a long list of drugs found at post mortem including methadone, gabapentin, fentanyl, MPA (NPS amphetamine-type), midazolam and a piperidine. She was prescribed methadone, pregabalin and quetiapine and had a history of chronic anxiety, bipolar disorder and was a known injecting drug user.

Several cases suggested there was some intent to the overdose but rarely were these cases recorded as suicide. The case of an 18-year-old who lived with her partner, was given an open verdict after cause of death given as poisoning by tramadol and oxycodone. Post mortem also found diazepam and phenazepam, a long-acting designer benzodiazepine, although she was prescribed chlorpromazine, tramadol and diazepam. The narrative included evidence of suicide:

“No signs of disturbance, with large amount of medications, some empty packets and several loose tablets found within bedside cupboard and on bed. Note found suggesting possible intention to end life. Smoked and drank large amounts of alcohol. Known drug abuser, mainly cannabis.”

Another young woman (aged 21) was also given an open verdict after being:

“Found after seen to take excessive quantities of prescribed medication the evening before death. Sharps bin with syringes and needles and crystal meth use was found at scene. She was found to have used 4-MEC, methadone, diazepam and codeine, being prescribed temazepam, diazepam, co-drydamol and propranolol.”

However, an older woman (aged 40) was given a verdict of suicide after taking an overdose of “unspecified medication”. She was reported to have a history of multiple overdoses and had access to fluoxetine, pregabalin, MPA (NPS amphetamine-type) diazepam and other conventional benzodiazepines. Only the fluoxetine, mirtazapine and diazepam were reported at post mortem among her prescribed medication, in addition to the MPA, mephedrone and cannabis were also found at post mortem.

One 18-year-old was also given a verdict of suicide without leaving a note but had texted a parent prior to taking an overdose of alcohol, diazepam and mephedrone and then hanging herself. The coroner reported that the drug use had impacted on her mental state (rather than the reverse) and she had been rejected by her boyfriend that evening at a club.

### Female NPS Related Deaths Not Associated With Methadone or Benzodiazepines

Examination of these cases revealed a predominance of younger deaths associated with recreational social use and with less polydrug use. Where cases were older, use appeared not to be linked with social activities; where the person was found dead or unwell whilst having used alone.

Typical examples of younger deaths included a 21-year-old:

“found at her home address lifeless. She had been out drinking with friends and is believed to have taken cocaine, bubble (mephedrone), amphetamine and alcohol.”

And similarly, a 20-year-old:

“Deceased with boyfriend and another friend, bought amphetamine, went to [outdoor leisure setting] where consumed it. Went back by car to her flat, was sick, said was cold and wanted to lie down. Condition worsened early hours. Boyfriend called ambulance [] put on ventilator but suffered liver problems.”

The deaths among the teens and those in their twenties have similar details, having been to a party or a friend's house, and taken either a single NPS, or mixed with alcohol. Occasionally there are two NPS substances reported as implicated, or an NPS and, commonly, an amphetamine. However, it is unclear if the NPS and amphetamine had been contained in a single tablet. None of these younger cases had prescribed medication reported or any history of mental illness. They were also often taken to hospital, having been with others at the time.

Older deaths without methadone or benzodiazepines typically had polydrug use as a feature. One 43-year-old died from coronary thrombosis after taking a synthetic cannabinoid (Black Mamba) with no other drug, however, she was prescribed buprenorphine, amitriptyline and codeine which were found at post mortem. There were several cases of older deaths where opioids were implicated, and this also differed markedly from younger deaths. One 42-year-old woman was found dead at home having taken over the counter tramadol and 4-MEC, a methcathinone. This woman had no known history of drug misuse and the verdict given was “non-dependent abuse of drugs”. There may have been other evidence available to the coroner to indicate the death was not intentional, however, the lethality of many NPS in circulation prior to 2016 was not generally accepted or understood by users, resulting in a spate of accidental overdoses and deaths.

## Discussion

The qualitative differences between methadone/benzodiazepine deaths among female NPS cases suggests different patterns of use by age which should be explored further. Older women also appear to be more likely to have a history of heroin use, regardless of this not showing up in prescribed methadone as a proxy variable for opiate addiction. Methadone is clearly available for illicit use among these cases, but prescribed medications for older women also commonly include anti-depressants, indicating some degree of psychological problems. It is a concern to see how often women whose deaths are associated with polydrug use, have prescribed medications likely to be interactive with substance misuse, for instance codeine-based medications.

Younger women's deaths without methadone or benzodiazepines implicated are less associated with regular or addictive drug use. The NPS deaths here clearly illustrate the lethality of the new substances, perhaps regarded as harmless due to being “legal highs” or associated with party drugs such as ecstasy.

By focusing on female NPS deaths, this analysis has gone some way in reducing the overshadowing effect of opiate deaths that otherwise dominates drug-related deaths research, and given more focus to the different profile that female deaths may present. Additionally, the qualitative examination of circumstances of death afforded by coronial narrative notes starts to give attention to female drug-death profiles.

### Greater Gender Homogeneity Among NPS Deaths

The comparison of NPS and non-NPS demographic and outcome associations highlights that NPS users may be more homogenous by gender than deaths where “conventional” illicit drugs are being used, such as opiates and cocaine. This effect may be associated with the younger age group that have typically used NPS, especially when NPS were more available to occasional and recreational users up to their outlawing in the UK by 2016.

As is typical for most drug-using cohorts, during 2007–2016 NPS use, there was reportedly a high prevalence of male users (19) and these substances tended to appeal to younger people (20). Populations found to be at particular risk of NPS mortality from database analysis in the UK were club attendees, men who have sex with men, prisoners and mentally ill inpatients, the homeless and people with eating disorders (21). A Swedish study of NPS use has shown how the gender gap reduced during the period of growth in use of NPS, where, in 2010, 20% of users were female, becoming nearer 30% by 2015, and average age of users had increased from 22 to 29 years (22). Our findings indicate that typical differences found in all drug-related deaths are not significant among deaths where NPS is implicated. What our findings suggest is that comparison between NPS deaths and non-NPS deaths indicate differences in usage and demographics other than gender. People dying following NPS use during this period were more likely to be younger, using fewer polydrug combinations, and more likely to die away from home than opiate users. This pattern may be more indicative of recreational use that has less gender difference than habitual use or addiction. Our examination of death circumstances also suggests NPS overdoses may more often occur in company than opiate overdoses. Therefore, where NPS have been used, these differences are less likely to be accounted by gender than other demographic or behavioral differences than would be typical for opiate deaths.

### Age Differences in Female Deaths

Older and more established drug-using women were found by Ataiants et al. (23) to report incidents of overdose when they had experienced stressful events or emotional disturbance, leading to atypical and more hazardous drug use, or change in circumstance such as leaving prison. Our analysis of circumstances surrounding a NPS death indicates a possible age difference among women and a different risk profile in that, older women using NPS may be more likely to be established drug users, familiar with accessing multiple illicit drugs, including methadone and over-the-counter opiates, and using hazardously when the death occurred. They were also typically at home on their own and not be taken to hospital. The population of injecting drug users in the UK is aging, encountering health problems and presenting with complex needs (24) and our evidence from older women supports this in demonstrating cases where social and health problems may be a feature of the person's circumstances. In this, women are likely to be facing similar global issues to men in their drug use histories, however we have not yet analyzed cases of male circumstances to suggest where differences may exist.

Younger NPS fatalities in this period appear to show more naïve usage, less polydrug use, and more likely to be using with friends and recreationally. A high proportion of the NPS deaths were associated with mephedrone, a cathinone shown to be particularly hazardous, but particularly popular as a club drug (25). The deaths where methadone and/or diazepam were implicated were also more typically associated with deaths of older women, who also tended to have social problems such as unstable living arrangement, and health problems. These deaths may reflect the spread of NPS from recreational use to the established drug-using populations and to more deprived locations [i.e., (26)], and to populations where polydrug use and self-medication is a more common behavior (27).

## Summary

There are very few studies that specifically examine gender difference in drug use *per se*, without targeting specific populations such as sex workers or focus on treatment outcomes, and there are none to our knowledge that examine female NPS deaths. Our key finding is that NPS deaths show reduced gender differences in demographics or outcomes than among drug-related deaths generally, indicating greater homogeneity between genders during a period of time when NPS were readily available to recreational users. As these users are more likely to be younger than the general population of drug users, age may be a factor in gender difference whereby gender divergence occurs among older drug users with longer drug use histories. Where NPS were used by older women, these cases typically involved greater polydrug use, including both illicit and over-the-counter drugs.

## Conclusion and Recommendations

Further studies that analyse drug deaths and drug use patterns by gender are needed to better understand vulnerability to drug mortality, particularly for different age groups who are typically using different drugs in different circumstances. An examination of patterns of NPS use by age and gender would be informative of risk from these substances specifically, especially where opiates are not a key feature of the cause of death or pattern of use.

## Limitations

This study isolated a core population in a time when NPS were largely unregulated and therefore only studied cases up to 2017, and does not reflect drug use patterns currently. Post mortems at that time also would not always include tests for NPS and some cases where NPS was a feature may have been excluded. We used “drugs implicated”, rather than “found at post mortem” as a more accurate indicator of the substances responsible, but this is not to imply the substance is a direct cause of death. For example, deaths included asphyxiation or other physical trauma. Additionally, mephedrone was made illegal in Europe in December 2010 which we found resulted in a brief reduction in NPS deaths in 2011. As the death rate returned to previous levels within 1 year, the dataset may not have been impacted significantly by this contextual change.

The dataset comprises only 76 NPS female cases, making gender comparison analysis underpowered, therefore these findings are indicative and require further analysis with a larger cohort and greater statistical power.

## Data Availability Statement

The data analyzed in this study is subject to the following licenses/restrictions: Privately owned data. Requests to access these datasets should be directed to hclaridg@sgul.ac.uk.

## Author Contributions

LW wrote the manuscript, contributed to analyses, and data collation. XS conducted the analysis and contributed to the reporting of findings. SC collated original data, contributed to the construction of the dataset, and the reporting of findings. CG supervised original data collection and formatting. All authors contributed to the article and approved the submitted version.

## Conflict of Interest

The authors declare that the research was conducted in the absence of any commercial or financial relationships that could be construed as a potential conflict of interest.

## Publisher's Note

All claims expressed in this article are solely those of the authors and do not necessarily represent those of their affiliated organizations, or those of the publisher, the editors and the reviewers. Any product that may be evaluated in this article, or claim that may be made by its manufacturer, is not guaranteed or endorsed by the publisher.
